# Risks of neonatal anomalies and obstetric complications in 7378 singleton births after frozen–thawed and fresh embryo transfers in Japan: An analysis using doubly robust estimation

**DOI:** 10.1002/rmb2.12623

**Published:** 2025-01-23

**Authors:** Sanae Terada, Toshihiro Habara, Ryo Terada, Toshiharu Mitsuhashi, Ryuhei So, Nanako Yoshioka, Yumi Masumoto, Yukiko Kosaka, Rei Hirata, Nobuyoshi Hayashi

**Affiliations:** ^1^ Reproductive Medicine Department Okayama Couple's Clinic Okayama Japan; ^2^ Terada Naika Clinic Okayama Japan; ^3^ Okayama University Hospital Center for Innovative Clinical Medicine Okayama Japan; ^4^ Okayama Psychiatric Medical Center Okayama Japan

**Keywords:** ART, fresh embryo transfer, frozen–thawed embryo transfer, neonatal anomalies, obstetric complications

## Abstract

**Purpose:**

To compare risks of neonatal anomalies and obstetric complications among frozen–thawed embryo transfer (FET), fresh embryo transfer (FreshET), and non‐assisted reproductive technology (non‐ART) treatments in infertile women.

**Methods:**

This retrospective cohort study analyzed 7378 singleton births (2643 non‐ART, 4219 FET, 516 FreshET) from 2013 to 2022. Outcomes were compared using inverse probability weighting regression adjustment, with adjustment for maternal factors.

**Results:**

After adjustment, the risk of neonatal anomalies did not differ significantly between FET and non‐ART, or FreshET and non‐ART. FET was associated with increased risks of obstetric complications compared with non‐ART, including placenta accreta (adjusted risk difference [ARD] 3.61%, 95% CI 2.95–4.28), placenta previa (ARD 0.55%, 95% CI 0.14–0.96), postpartum hemorrhage (ARD 7.08%, 95% CI 6.03–8.13), gestational hypertension (ARD 3.57%, 95% CI 2.47–4.68), gestational diabetes (ARD 0.96%, 95% CI 0.17–1.75), and preterm birth (ARD 2.13%, 95% CI 1.23–3.02). FET also showed higher risk of high birth weight (ARD 0.97%, 95% CI 0.42–1.52). FreshET showed no significant differences in obstetric complications.

**Conclusions:**

While the risk of neonatal anomalies did not differ among treatments, FET was associated with increased obstetric complication risks. These findings underscore the need for careful management of FET pregnancies and further research to improve treatment protocols.

## Graphical abstract

This retrospective cohort study compared neonatal anomalies and obstetric complications among 7378 singleton births resulting from frozen–thawed embryo transfer (FET), fresh embryo transfer (FreshET), and non‐assisted reproductive technology (non‐ART) treatments. While the risk of neonatal anomalies did not differ significantly among the three groups, FET was associated with increased risks of several obstetric complications compared to non‐ART, including placenta accreta, postpartum hemorrhage, and gestational hypertension. These findings underscore the need for careful management of FET pregnancies and further research to improve treatment protocols.

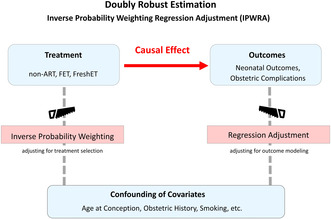

## 1 INTRODUCTION

The use of assisted reproductive technology (ART) has increased worldwide and plays a significant role in reproductive medicine. ART is responsible for 1%–7% of all births in developed countries, highlighting its growing significance in addressing infertility challenges.^1^, ^2^ The Japan Society of Obstetrics and Gynecology (JSOG) has collected cycle‐based data on ART in an online registry. According to the registry, 498 140 ART cycles were reported in 2021, resulting in 69 797 live births,^3^ underscoring the importance of evaluating their effects on neonatal and maternal health to ensure the safety of these treatments.

Some evidence suggests an increased risk of neonatal anomalies in children born through ART, which may be related to the ART procedures themselves or to the underlying parental infertility.^4^, ^5^, ^6^, ^7^ However, the comparative effects of frozen–thawed embryo transfer (FET) and fresh embryo transfer (FreshET) on neonatal anomalies remain unclear. In addition, FET is associated with several obstetric complications, including gestational hypertension, postpartum hemorrhage, and increased birth weight,^8^, ^9^ which can affect both maternal and neonatal health.

This study aimed to evaluate the safety profiles of FET and FreshET in comparison with non‐assisted reproductive technology (non‐ART) infertility treatments as a reference group among infertile women, focusing on the risks of neonatal anomalies and obstetric complications. By employing inverse probability weighting regression adjustment (IPWRA), we sought to provide a detailed comparative analysis, thereby contributing valuable insights into the field of reproductive medicine.

## 2 MATERIALS AND METHODS

### 2.1 Study design and settings

This retrospective observational study was conducted at a Japanese infertility center (Okayama Couple's Clinic, Okayama, Japan), focusing on singleton births between January 2013 and December 2022 resulting from infertility treatments. Our clinic specializes in fertility treatment, and coordinates with childbirth‐equipped hospitals for delivery management. Our clinic follows the guidelines and standard practices of fertility treatments in Japan.

### 2.2 Participants

**FIGURE 1:**
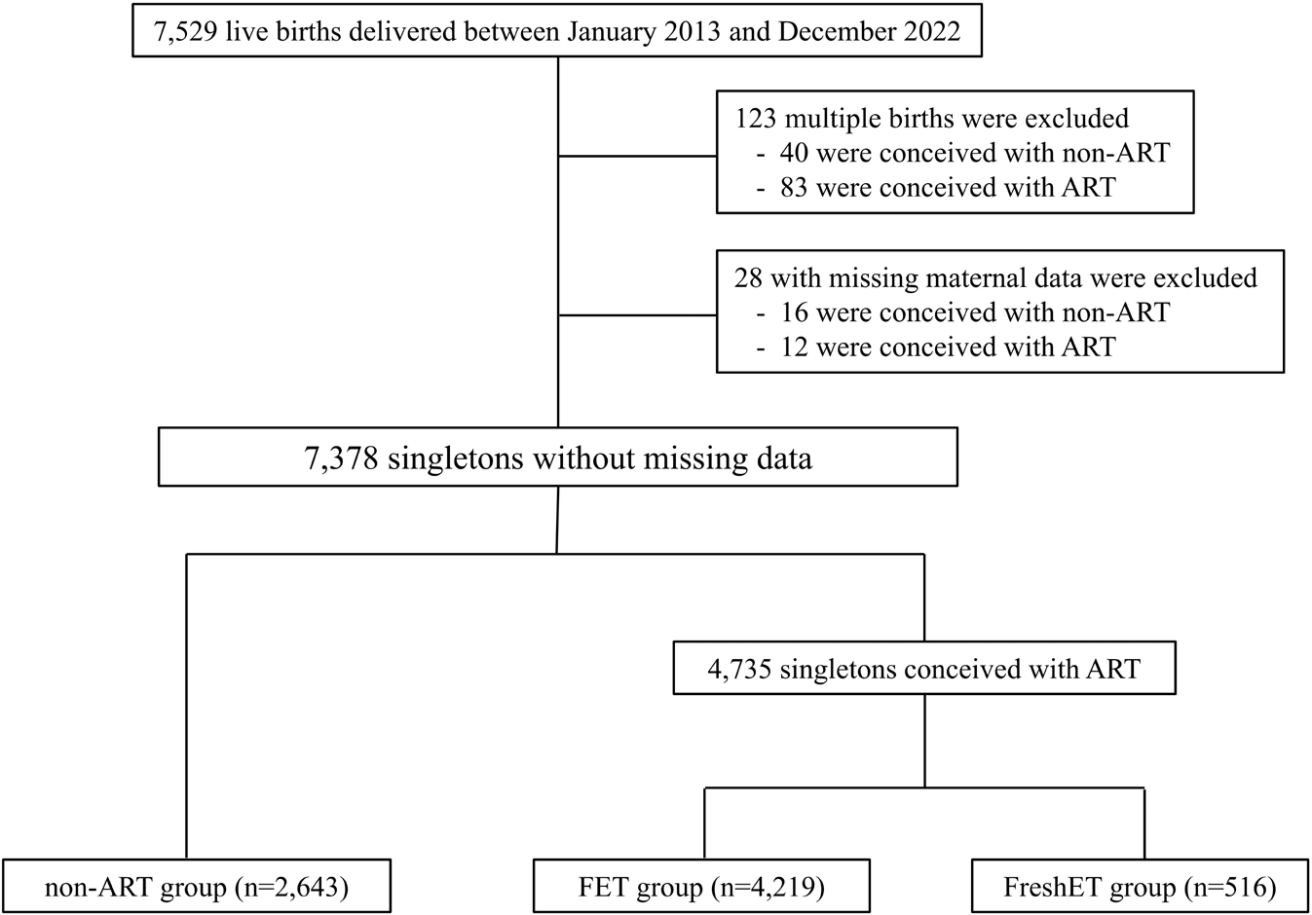
Flowchart of the study populations. ART, assisted reproductive technology; FET, frozen–thawed embryo transfer; FreshET, fresh embryo transfer.

We included all singleton births between January 2013 and December 2022 resulting from infertility treatments at our clinic. Patients who conceived through non‐ART methods such as controlled ovarian stimulation, timed intercourse, intrauterine insemination, or subsequent to routine infertility examinations were categorized into the non‐ART group, which served as a reference group for comparison with the FET and FreshET cohorts regarding neonatal anomalies and obstetric complications. To ensure the reliability of our analyses, we excluded cases of multiple births, pregnancies that did not result in live births, and cases lacking complete maternal data, including age at conception, pregnancy history, delivery history, miscarriage history, cesarean section history, body mass index, and smoking history (Figure 1).

### 2.3 Data collection and variables

Comprehensive data on neonatal outcomes and obstetric complications were routinely obtained from referral hospitals. We actively obtained data from these hospitals through letters, faxes, and phone calls when necessary to ensure data completeness. Data were managed using FileMaker Pro (FileMaker, Inc., Santa Clara, CA, USA). All patient information was anonymized and de‐identified prior to analysis to protect privacy and ensure confidentiality.

The neonatal outcomes tracked included neonatal anomalies identified at perinatal facilities at birth, high birth weight (≥4000 g), and low birth weight (<2500 g). Obstetric complications were identified using a standardized coordination sheet shared between our clinic and the referral hospitals. The sheet includes checkboxes for predefined complications, such as placenta accreta, placenta previa, postpartum hemorrhage, gestational hypertension, gestational diabetes, and placental abruption. It also provides fields for reporting gestational age at delivery, allowing for the identification of preterm births (birth before 37 weeks of gestation), and a free‐text field for any additional complications, ensuring comprehensive data capture. Neonatal anomalies were reported as free‐text responses by healthcare providers at referral hospitals. These reports were then retrospectively categorized by our research team according to the International Classification of Diseases 10th revision.

### 2.4 Statistical analysis

Statistical analyses were performed using Stata software (version 17.0; Stata Corp, College Station, TX, USA). Maternal background factors of the FET and FreshET groups were compared with those of the non‐ART group. Continuous variables were analyzed using t‐tests, whereas categorical variables were examined using Fisher's exact test, with a significance threshold of *p* < 0.05.

Given the retrospective nature of this study, we included all available maternal background factors as covariates in the analysis to control for potential confounding. These factors were selected based on their availability in the database and potential influence on the outcomes of interest.

We used the IPWRA to compare neonatal outcomes and obstetric complications.^10^, ^11^ IPWRA is a combined inverse‐probability weighting and regression adjustment method, and is referred to as a doubly robust estimator. That is, weights calculated by the propensity score remove the confounding of covariates for the treatment. Regression adjustment, on the other hand, eliminates covariate confounding for outcomes. The IPWRA method allows for an unbiased estimation of the average treatment effect, provided that either the propensity score model or the outcome regression model is specified correctly. The propensity scores were calculated using a multinomial logistic regression model with treatment (non‐ART/FET/FreshET) as the response variable and confounding factors as the explanatory variables. The inverse probability weight (IPW) was calculated as the inverse of the probability of receiving the actual treatment for each subject. In the regression adjustment, IPW was used as the weight, while the expected outcomes for each treatment group were predicted using the confounders and the regression model. The effects of treatment on the outcomes were determined by subtracting the predicted average outcome for the non‐ART group from the FET or FreshET group.

We calculated potential outcomes using all maternal background factors as covariates in both the treatment (propensity score) and outcome models (regression adjustment). Risk differences between the FET and non‐ART groups and between the FreshET and non‐ART groups, along with their 95% confidence intervals, were calculated. Potential outcomes and their differences were presented as risks and risk differences.

In addition, sensitivity analysis was performed on the results of each analysis. E‐values were computed for the adjusted risk differences in the primary outcomes to evaluate the robustness of our results against unmeasured confounding. An E‐value indicates the minimum magnitude of association that an unmeasured confounder would require with both the treatment and outcome to completely nullify the observed effect, serving as an indicator of the study's resilience to potential unmeasured biases.

For the analysis of neonatal anomalies, the number of individual cases of anomalies in the non‐ART, FET, and FreshET groups was counted according to the ICD‐10 classification, and their proportions were calculated. Risk differences and 95% confidence intervals were then computed for the FET and FreshET groups, respectively, using the non‐ART group as a reference, adjusting for all maternal factors using a linear probability model.^12^

## 3 RESULTS

### 3.1 Flowchart of the study populations

From January 2013 to December 2022, 7529 live births were recorded at our clinic following infertility treatment. After excluding 123 cases of multiple births and 28 cases with incomplete maternal data, we analyzed 7378 singleton births. These were divided into 2643 non‐ART, 4219 FET, and 516 FreshET cases (Figure 1).

### 3.2 Comparison of maternal background factors

Table 1 shows a comparison of maternal background factors. The FET group exhibited higher rates of pregnancy history (62.0% vs. 45.9%, *p* < 0.001), childbirth history (39.8% vs. 27.0%, *p* < 0.001), miscarriage history (40.6% vs. 30.9%, *p* < 0.001), and cesarean section history (10.2% vs. 4.0%, *p* < 0.001) compared to the non‐ART group. Additionally, the average age at conception was higher in the FET group (34.9 years vs. 32.6 years, *p* < 0.001), and the average gestational weeks were shorter (38.7 weeks vs. 38.8 weeks, p < 0.001). The FreshET group also showed a higher average age at conception than the non‐ART group (34.6 years vs. 32.6 years, *p* < 0.001), with no significant differences in the other factors.

**TABLE 1:** Comparison of maternal background factors.

	Non‐ART (*n* = 2643)	ART			
		FET (*n* = 4219)	*p*‐value	FreshET (*n* = 516)	*p*‐value
Pregnancy history, *n* (%)			**<0.001**		0.596
Yes	1212 (45.9)	**2615 (62.0)**		230 (44.6)	
No	1431 (54.1)	**1604 (38.0)**		286 (55.4)	
Childbirth history, *n* (%)			**<0.001**		0.173
Yes	713 (27.0)	**1681 (39.8)**		123 (24.0)	
No	1930 (73.0)	**2538 (60.2)**		392 (76.0)	
Miscarriage history, *n* (%)			**<0.001**		0.497
Yes	816 (30.9)	**1715 (40.6)**		151 (29.4)	
No	1827 (69.1)	**2504 (59.4)**		365 (70.7)	
Cesarian section history, *n* (%)			**<0.001**		0.398
Yes	106 (4.0)	**430 (10.2)**		25 (4.8)	
No	2537 (96.0)	**3789 (89.8)**		491 (95.2)	
Smoking			0.115		0.727
Yes	119 (4.5)	157 (3.7)		21 (4.1)	
No	2524 (95.5)	4062 (96.3)		495 (95.9)	
Age at conception, mean ± SD	32.6 ± 4.2	**34.9** ± **4.3**	**<0.001**	**34.6** ± **4.0**	**<0.001**
BMI, mean ± SD	21.0 ± 3.2	21.0 ± 3.0	0.795	21.1 ± 3.2	0.428
Gestational weeks, mean ± SD	38.8 ± 1.7	**38.7** ± **2.2**	**<0.001**	38.8 ± 1.6	0.790

*Note*: The FET and FreshET groups were compared with the non‐ART group, with the non‐ART group as a reference. Differences between the groups were evaluated using the t‐test for continuous variables and Fisher's exact test for categorical variables. Variables with significant difference are indicated in bold.

Abbreviations: ART, assisted reproductive technology; BMI, body mass index; FET, frozen–thawed embryo transfer; FreshET, fresh embryo transfer; SD, standard deviation.

### 3.3 Distribution of propensity scores and covariate balancing

**FIGURE 2:**
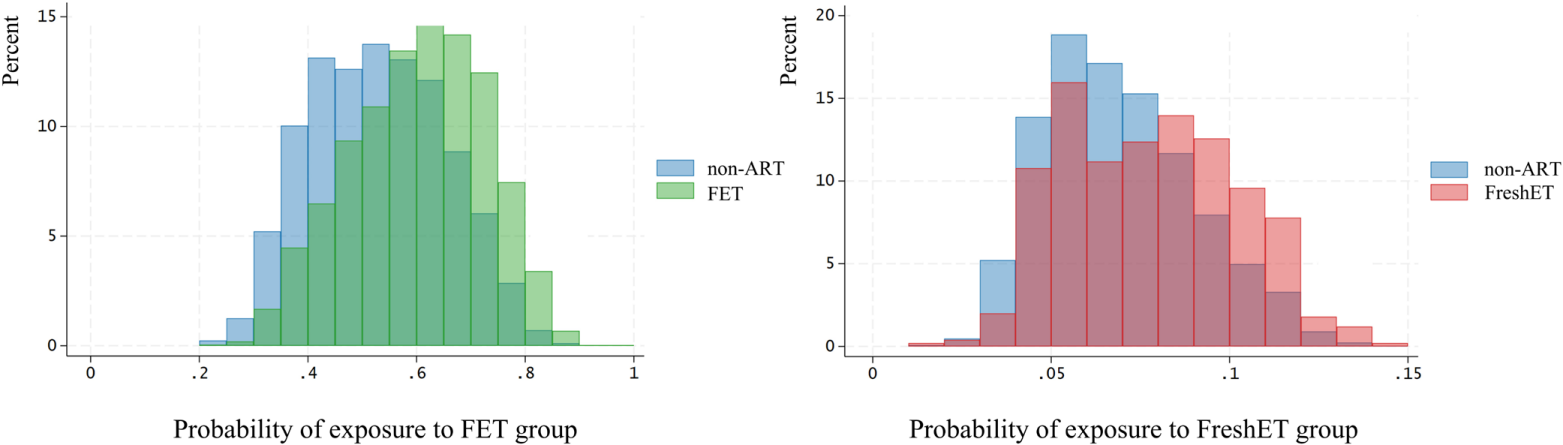
Distribution of propensity scores between non‐ART and FET (left) and between non‐ART and FreshET (right) ART, assisted reproductive technology; FET, frozen–thawed embryo transfer; FreshET, fresh embryo transfer. Propensity scores are largely overlapped, indicating a balanced probability of exposure in each treatment group.

Figure 2 presents the distribution of the propensity scores. The propensity scores were well balanced across the groups, as indicated by the largely overlapping weighted propensity scores, ensuring balanced exposure probabilities in each treatment group. Table 2 shows the standardized mean differences and variance ratios used to evaluate the balance of the covariates. The standardized mean differences and variance ratios between the groups approached the ideal values of zero and one, respectively, indicating well‐balanced post‐weighting covariates.^13^, ^14^

**TABLE 2:** Covariate balance summary.

		Standardized mean differences		Variance ratios	
		Raw	Weighted	Raw	Weighted
FET vs. non‐ART	Pregnancy history	0.328	−0.006	0.949	1.001
	Childbirth history	−0.275	0.008	1.217	0.995
	Miscarriage history	0.213	0.004	1.484	1.132
	Cesarian section history	0.242	0.007	2.377	1.022
	Smoking	−0.039	−0.004	0.833	0.982
	Age at conception	0.537	0.008	1.057	1.077
	BMI	−0.006	0.004	0.862	0.926
	Gestational weeks	−0.082	−0.002	1.653	1.453
	Average of absolute values	0.214	0.005	1.304	1.074
FreshET vs. non‐ART	Pregnancy history	−0.026	0.014	0.997	0.997
	Childbirth history	0.068	0.010	0.928	0.994
	Miscarriage history	−0.001	0.035	1.091	1.223
	Cesarian section history	0.041	0.004	1.199	1.014
	Smoking	−0.021	0.017	0.909	1.081
	Age at conception	0.483	0.023	0.910	0.915
	BMI	0.038	−0.017	0.972	0.982
	Gestational weeks	−0.013	−0.030	0.902	0.938
	Average of absolute values	0.088	0.018	0.988	1.018

*Note*: Balance evaluation of covariates between the non‐ART group and each ART group. The standardized mean differences and variance ratios between the groups approached ideal values of zero and one, respectively, after weighting (weighted) compared with those before weighting (raw).

Abbreviations: ART, assisted reproductive technology; BMI, body mass index; FET, frozen–thawed embryo transfer; FreshET, fresh embryo transfer.

### 3.4 Comparison of neonatal outcomes

The risk differences for neonatal outcomes between the FET and non‐ART groups, and between the FreshET and non‐ART groups, as well as the risk values for each group, are shown in Table 3. Crude analysis initially revealed a significant risk difference for neonatal anomalies between the FET and non‐ART groups (crude risk difference 0.74%, 95% CI 0.09–1.38). After adjustment, this difference was attenuated and no longer statistically significant (ARD 0.68%, 95% CI 0.02–1.39). The adjusted risks for neonatal anomalies in the non‐ART, FET, and FreshET groups were 1.63%, 2.31%, and 1.93%, respectively. The FreshET group did not exhibit significant risk differences for neonatal anomalies in either the crude or adjusted analyses. Regarding birth weight, the FET group had a higher risk of high birth weight than the non‐ART group (ARD 0.97%, 95% CI 0.42–1.52), while no significant differences were observed for low birth weight between both the FET and FreshET groups and the non‐ART group.

**TABLE 3:** Risk comparison of neonatal outcomes between the non‐ART, FET, and FreshET groups.

Outcomes	Non‐ART (*n* = 2643)	FET (*n* = 4219)	FreshET (*n* = 516)	Analysis	Comparison	Risk difference (%)	95% CI	*p*‐value	*E*‐value
Neonatal anomalies	40 (1.51)^a^	95 (2.25)^a^	10 (1.94)^a^	Crude	FET vs. non‐ART	**0.74**	**0.09 to 1.38**	**0.025**	**2.34**
					FreshET vs. non‐ART	0.42	−0.85 to 1.70	0.515	1.88
				Adjusted	FET vs. non‐ART	0.68	−0.02 to 1.39	0.058	2.19
					FreshET vs. non‐ART	0.30	−1.03 to 1.63	0.659	1.65
Low birth weight (<2500 g)	212 (8.02)	349 (8.27)	53 (10.27)	Crude	FET vs. non‐ART	0.25	−1.08 to 1.58	0.711	1.21
					FreshET vs. non‐ART	2.25	−0.57 to 5.07	0.117	1.88
				Adjusted	FET vs. non‐ART	−0.63	−1.92 to 0.66	0.340	1.37
					FreshET vs. non‐ART	1.66	−0.95 to 4.27	0.212	1.67
High birth weight (≥4000 g)	22 (0.83)	72 (1.71)	5 (0.97)	Crude	FET vs. non‐ART	**0.87**	**0.35 to 1.40**	**0.001**	**3.52**
					FreshET vs. non‐ART	0.14	−0.78 to 1.05	0.769	1.60
				Adjusted	FET vs. non‐ART	**0.97**	**0.42 to 1.52**	**0.001**	**3.85**
					FreshET vs. non‐ART	0.08	−0.82 to 0.98	0.856	1.44

*Note*: Significant differences in risk are indicated in bold.

Abbreviations: ART, assisted reproductive technology; CI, confidence interval; FET, frozen–thawed embryo transfer; FreshET, fresh embryo transfer.

^a^ Data are presented as n (crude risk %), where n is the number of cases and crude risk % is calculated as (number of cases/total group size) × 100.

E‐values, which indicate the robustness of our findings against unmeasured confounding factors, were calculated for the outcomes. For neonatal anomalies, the E‐values for the crude and adjusted analyses in the FET group were 2.34 and 2.19, respectively. The E‐value for high birth weight in the FET group was 3.85. We further investigated the specific types of neonatal anomalies in each group (Table 4).

**TABLE 4:** Classification of neonatal anomalies and risk comparison between the non‐ART, FET, and FreshET groups.

Category (ICD‐10 code)	Non‐ART (*n* = 2643)	FET (*n* = 4219)	FreshET (*n* = 516)	Comparison	Adjusted risk difference (%)	95% CI	*p*‐value
Congenital malformations, deformations, and chromosomal abnormalities (Q00‐99)							
Nervous system (Q00‐07)	1 (0.038)^a^	4 (0.095)^a^	1 (0.194)^a^	FET vs. non‐ART	1.81	−6.09 to 9.70	0.652
				FreshET vs. non‐ART	7.19	−7.83 to 22.20	0.345
Eyelid, lacrimal apparatus and orbit (Q10‐18)	5 (0.190)	4 (0.095)	0 (0.000)	FET vs. non‐ART	−7.96	−16.51 to 0.60	0.068
				FreshET vs. non‐ART	−15.84	−32.12 to 0.43	0.056
Circulatory system (Q20‐28)	5 (0.190)	18 (0.427)	5 (0.969)	FET vs. non‐ART	6.86	−8.36 to 22.08	0.374
				FreshET vs. non‐ART	**40.06**	**11.12 to 69.00**	**0.007**
Respiratory system (Q30‐34)	1 (0.038)	2 (0.047)	0 (0.000)	FET vs. non‐ART	−0.45	−5.96 to 5.05	0.871
				FreshET vs. non‐ART	−2.01	−12.48 to 8.46	0.705
Cleft palate/lip (Q35‐37)	2 (0.076)	7 (0.166)	0 (0.000)	FET vs. non‐ART	1.09	−8.25 to 10.43	0.818
				FreshET vs. non‐ART	−9.97	−27.74 to 7.79	0.269
Digestive system (Q38‐45)	5 (0.190)	9 (0.213)	0 (0.000)	FET vs. non‐ART	−2.29	−13.82 to 9.25	0.696
				FreshET vs. non‐ART	−8.52	−30.46 to 13.42	0.444
Genital organs (Q50‐56)	1 (0.038)	4 (0.095)	1 (0.194)	FET vs. non‐ART	1.89	−5.92 to 9.69	0.634
				FreshET vs. non‐ART	8.09	−6.75 to 22.93	0.283
Urinary system (Q60‐64)	2 (0.076)	11 (0.261)	0 (0.000)	FET vs. non‐ART	9.19	−1.35 to 19.74	0.087
				FreshET vs. non‐ART	1.27	−18.78 to 21.32	0.900
Musculoskeletal system (Q65‐79)	7 (0.265)	15 (0.356)	1 (0.194)	FET vs. non‐ART	−0.40	−14.57 to 13.76	0.955
				FreshET vs. non‐ART	−11.57	−38.51 to 15.36	0.397
Other congenital malformations (Q80‐89)	4 (0.151)	4 (0.190)	0 (0.000)	FET vs. non‐ART	−6.47	−15.34 to 2.41	0.152
				FreshET vs. non‐ART	−10.86	−27.73 to 6.02	0.205
Chromosomal abnormalities (Q90‐99)	5 (0.190)	8 (0.190)	0 (0.000)	FET vs. non‐ART	−6.58	−17.86 to 4.70	0.251
				FreshET vs. non‐ART	−2.41	−23.86 to 19.04	0.824
Endocrine, nutritional and metabolic disease (E00‐E90)							
Metabolic disorders (E70‐E90)	1 (0.038)	1 (0.024)	1 (0.194)	FET vs. non‐ART	−1.65	−6.26 to 2.96	0.481
				FreshET vs. non‐ART	−3.36	−12.13 to 5.41	0.450
Adrenogenital disorders (E25)	0 (0.000)	1 (0.024)	0 (0.000)	FET vs. non‐ART	1.03	−2.23 to 4.29	0.531
				FreshET vs. non‐ART	−0.26	−6.46 to 5.94	0.934
Diseases of the ear and mastoid process (H60‐H95)							
Other disorders of ear (H90‐95)	1 (0.038)	2 (0.047)	0 (0.000)	FET vs. non‐ART	−0.01	−5.61 to 5.60	0.998
				FreshET vs. non‐ART	−3.06	−13.73 to 7.60	0.571
Neoplasms (C00‐D48)							
Neoplasm of uncertain behavior (D48)	0 (0.000)	1 (0.024)	0 (0.000)	FET vs. non‐ART	1.33	−1.91 to 4.56	0.419
				FreshET vs. non‐ART	0.78	−5.38 to 6.93	0.803
Others							
Multiple defects	0 (0.000)	3 (0.071)	1 (0.194)	FET vs. non‐ART	2.60	−3.59 to 8.78	0.407
				FreshET vs. non‐ART	10.48	−1.28 to 22.24	0.080

*Note*: Codes in parentheses indicate International Classification of Diseases 10th revision (ICD‐10). Significant differences in risk are indicated in bold.

Abbreviations: ART, assisted reproductive technology; CI, confidence interval; FET, frozen–thawed embryo transfer; FreshET, fresh embryo transfer.

^a^ Data are presented as *n* (crude risk %), where n is the number of cases and crude risk % is calculated as (number of cases/total group size) × 100. Three decimal places are used due to the rarity of some conditions.

Table 4 compares the classification of neonatal anomalies in each group and the risk differences between the FET and non‐ART groups, and between the FreshET and non‐ART groups. After adjusting for all maternal factors, the FreshET group showed a significantly increased risk of congenital malformations of the circulatory system compared with the non‐ART group. However, no other specific neonatal anomalies were associated with a significantly increased risk.

### 3.5 Comparison of obstetric complications

The risk differences in obstetric complications between the non‐ART and ART groups as well as the risk values for each group are shown in Table 5. The FET group was associated with higher risks of several obstetric complications, including placenta accreta (ARD 3.61%, 95% CI 2.95–4.28), placenta previa (ARD 0.55%, 95% CI 0.14–0.96), postpartum hemorrhage (ARD 7.08%, 95% CI 6.03–8.13), gestational hypertension (ARD 3.57%, 95% CI 2.47–4.68), gestational diabetes (ARD 0.96%, 95% CI 0.17–1.75), and preterm birth (ARD 2.13%, 95% CI 1.23–3.02). In contrast, the FreshET group did not show significant adjusted risk differences for any obstetric complications compared with the non‐ART group. The E‐values for outcomes with increased adjusted risk differences in the FET group were as follows: placenta accreta 16.23, postpartum hemorrhage 8.88, placenta previa 3.83, gestational hypertension 3.60, preterm birth 2.20, and gestational diabetes 2.19.

**TABLE 5:** Risk comparison of obstetric complications between the non‐ART, FET, and FreshET groups.

Outcomes	Non‐ART (*n* = 2643)	FET (*n* = 4219)	FreshET (*n* = 516)	Analysis	Comparison	Risk difference (%)	95% CI	*p*‐value	*E*‐value
Placenta accreta	13 (0.49)^a^	180 (4.27)^a^	4 (0.78)^a^	Crude	**FET vs. non‐ART**	**3.77**	**3.11 to 4.44**	**<0.001**	**16.83**
					FreshET vs. non‐ART	0.28	−0.52 to 1.09	0.489	2.53
				Adjusted	**FET vs. non‐ART**	**3.61**	**2.95 to 4.28**	**<0.001**	**16.23**
					FreshET vs. non‐ART	0.42	−0.54 to 1.39	0.391	3.13
Placenta previa	12 (0.45)	43 (1.02)	6 (1.16)	Crude	**FET vs. non‐ART**	**0.57**	**0.17 to 0.96**	**0.005**	**3.92**
					FreshET vs. non‐ART	0.71	−0.25 to 1.67	0.148	4.56
				Adjusted	**FET vs. non‐ART**	**0.55**	**0.14 to 0.96**	**0.008**	**3.83**
					FreshET vs. non‐ART	0.87	−0.26 to 1.99	0.131	5.21
Postpartum hemorrhage	50 (1.89)	374 (8.86)	15 (2.91)	Crude	**FET vs. non‐ART**	**6.97**	**5.97 to 7.98**	**<0.001**	**8.84**
					FreshET vs. non‐ART	1.02	−0.52 to 2.55	0.196	2.44
				Adjusted	**FET vs. non‐ART**	**7.08**	**6.03 to 8.13**	**<0.001**	**8.88**
					FreshET vs. non‐ART	0.96	−0.64 to 2.56	0.239	2.37
Gestational hypertension	75 (2.84)	281 (6.66)	20 (3.88)	Crude	**FET vs. non‐ART**	**3.82**	**2.84 to 4.81**	**<0.001**	**4.13**
					FreshET vs. non‐ART	1.04	−0.74 to 2.82	0.253	2.07
				Adjusted	**FET vs. non‐ART**	**3.57**	**2.47 to 4.68**	**<0.001**	**3.60**
					FreshET vs. non‐ART	0.22	−1.49 to 1.94	0.798	1.34
Gestational diabetes	62 (2.35)	145 (3.44)	12 (2.33)	Crude	**FET vs. non‐ART**	**1.09**	**0.29 to 1.89**	**0.007**	**2.29**
					FreshET vs. non‐ART	−0.02	−1.44 to 1.40	0.978	1.10
				Adjusted	**FET vs. non‐ART**	**0.96**	**0.17 to 1.75**	**0.017**	**2.19**
					FreshET vs. non‐ART	−0.48	−1.69 to 0.72	0.434	1.85
Preterm birth	125 (4.73)	312 (7.40)	23 (4.46)	Crude	**FET vs. non‐ART**	**2.67**	**1.53 to 3.80**	**<0.001**	**2.50**
					FreshET vs. non‐ART	−0.27	−2.23 to 1.68	0.785	1.32
				Adjusted	**FET vs. non‐ART**	**2.13**	**1.23 to 3.02**	**<0.001**	**2.20**
					FreshET vs. non‐ART	−0.52	−2.06 to 1.03	0.514	1.47
Placental abruption	11 (0.42)	22 (0.52)	1 (0.19)	Crude	FET vs. non‐ART	0.11	−0.22 to 0.43	0.529	1.82
					FreshET vs. non‐ART	−0.22	−0.67 to 0.23	0.335	3.72
				Adjusted	FET vs. non‐ART	0.03	−0.31 to 0.37	0.864	1.33
					FreshET vs. non‐ART	−0.21	−0.76 to 0.33	0.447	3.16

Abbreviations: ART, assisted reproductive technology; CI, confidence interval; FET, frozen–thawed embryo transfer; FreshET, fresh embryo transfer.

^a^ Data are presented as n (crude risk %), where n is the number of cases and crude risk % is calculated as (number of cases/total group size) × 100. Significant differences in risk are indicated in bold.

## 4 DISCUSSION

The current study provides valuable insights into the safety of FET and FreshET compared to that of non‐ART treatments among infertile women. Crude analysis initially showed a higher risk of neonatal anomalies in the FET group than in the non‐ART group; however, this difference was attenuated and became nonsignificant after adjustment. The prevalence of neonatal anomalies in the general population varies across studies, ranging from approximately 1.5% to 3%.^15^, ^16^, ^17^, ^18^ In our study, the prevalence of neonatal anomalies was 1.63% and 2.31% in the non‐ART and FET groups, respectively. The adjusted risk difference of 0.68% (95% CI 0.02–1.39) between the non‐ART and FET groups in our study may not be large enough to dissuade couples struggling with infertility from considering FET. Therefore, it is crucial to develop safer ART methods for reproductive medicine practitioners. Understanding the source of this difference is essential for identifying potential areas for improvement in FET techniques and providing accurate information to patients regarding the risks and benefits of different treatment options.

While previous studies have reported that ART is associated with an increased risk of neonatal anomalies compared to spontaneous conception,^19^, ^20^, ^21^ few studies have directly compared the risk of neonatal anomalies between specific ART techniques, such as FET and FreshET, and non‐ART treatments among infertile women. Our study addresses this gap by providing a comprehensive analysis of the risk of neonatal anomalies in infertile patients undergoing non‐ART, FET, and FreshET treatments. A meta‐analysis including 45 cohort studies reported that the relative risk of neonatal anomalies for ART compared to those of spontaneous pregnancy was 1.32 (95% CI 1.24–1.42).^7^ However, the risk of neonatal anomalies in ART is thought to be related to both the ART procedures and the underlying infertility factors in the parents. By comparing infertile women undergoing different treatments, our study helps elucidate the specific effects of FET and FreshET on the risk of neonatal anomalies, while controlling for the influence of infertility itself. Previous studies from Finland and the United Kingdom that compared FET and FreshET found no major differences in neonatal anomaly rates between these two ART techniques,^22^, ^23^ but they did not include a non‐ART comparison group. Our study extends these findings by demonstrating that after adjusting for confounding factors, the risk of neonatal anomalies is not significantly different among infertile patients undergoing non‐ART, FET, and FreshET treatments, providing valuable insights into the relative safety of these approaches in the context of infertility.

Despite the overall lack of significant differences in the neonatal anomalies, our analysis of specific anomaly types revealed a potential area of concern. The risk of circulatory system anomalies was significantly higher in the FreshET group than in the non‐ART group. This finding is consistent with a previous report on increased congenital circulatory anomalies in FreshETs.^24^ Exposure of the transferred embryo to hormonal stimulation in the early stages of FreshET may have contributed to an increased risk of circulatory system anomalies. However, further research is needed to confirm this hypothesis and elucidate the underlying mechanisms.

Our study corroborates previous findings regarding the association between FET and an increased risk of obstetric complications. We found that FET was associated with higher rates of gestational hypertension, placenta accreta, and postpartum hemorrhage, which is consistent with previous studies.^9^, ^25^, ^26^ The high E‐values for placenta accreta and postpartum hemorrhage suggest that these findings are less likely to be affected by unmeasured confounding. For instance, the E‐value of 16.23 for placenta accreta indicates that an unmeasured confounder must have a risk ratio of at least 16.23 with both FET and placenta accreta to explain away the observed association, which is unlikely given the strengths of the known risk factors.

However, in contrast to previous meta‐analyses, our study found a higher risk of preterm birth associated with FET.^8^, ^27^ Furthermore, our study revealed an increased risk of gestational diabetes in the FET group, contradicting research suggesting that hormone replacement therapy in FET cycles lowers this risk.^28^ These discrepancies highlight the need for further investigation to identify the specific factors contributing to increased risk. The relatively low E‐values for preterm birth and gestational diabetes (2.20 and 2.19, respectively) indicated that these findings could be influenced by unobserved confounding factors, underscoring the importance of cautious interpretation. Future studies should focus on investigating the underlying mechanisms and potential confounders associated with obstetric complications of FET. Moreover, it is crucial for ART and perinatal care facilities to share information on the risks posed to mothers and neonates using different ART techniques to ensure appropriate care for both mothers and children.

Our study has several strengths that contribute to narrowing the knowledge gap in reproductive medicine. First, we employed the IPWRA method, which has rarely been used in previous studies. This novel approach allows for a more effective adjustment for confounding factors, enhancing the reliability of our findings. Second, our study focused on women with infertility and compared the outcomes of non‐ART, FET, and FreshET treatments within this specific population. This is particularly valuable because many fertility clinics specialize in ART and do not actively offer non‐ART treatments. Consequently, comparisons between these groups are less common in the existing literature. By including both ART and non‐ART treatments in our analysis, we provide a more comprehensive understanding of the relative risks associated with each approach in women with infertility.

However, our study has several limitations that should be considered when interpreting the results. A single‐facility setting may limit the generalizability of the findings to broader populations and settings. The outcomes observed in this study may have been influenced by factors specific to the facility, such as patient demographics and clinical practices, which could further affect the applicability of the results to other populations and healthcare settings. Additionally, the retrospective nature of this study may introduce the possibility of bias due to unmeasured confounding factors that cannot be entirely ruled out. Furthermore, our covariate selection was limited to the maternal background factors available in our database. We acknowledge that this approach may have failed to capture all relevant confounding factors, potentially introducing bias. To address this limitation, we incorporated E‐values into our analysis to demonstrate the potential impact of unmeasured confounders on our results.

Moreover, almost all our FET patients were transferred with hormone replacement cycles, which may limit the applicability of our findings to FET with natural cycle protocols. FET can be performed using either hormone replacement or natural cycles for endometrial preparation. Recent studies have shown that FET with hormone replacement cycles is associated with an increased risk of adverse obstetric and perinatal outcomes, including gestational hypertension, placenta accreta, and postpartum hemorrhage, compared to natural cycle FET.^28^, ^29^ While FET with hormone replacement cycles has been widely used because it allows for flexible scheduling of embryo transfer timing without relying on the patient's natural ovulation, the increased risk of obstetric complications has raised concerns regarding its selection in recent years. Given that 97.5% of our FETs were performed using hormone replacement cycles, our results may not be generalizable to FET using natural cycle protocols, which is a significant limitation of our study.

Regarding obstetric complications, our study was limited by the inability to include preeclampsia in the analysis, despite including gestational hypertension. This limitation was based on two main considerations. First, the diagnostic criteria for preeclampsia underwent significant changes during our study period (2013–2022), particularly with the 2020 revision of JSOG guidelines, which modified the diagnostic criteria from requiring both hypertension and proteinuria to including cases with organ dysfunction or uteroplacental dysfunction even without proteinuria. Second, our data collection system did not capture the detailed clinical data required for consistent preeclampsia classification (such as proteinuria levels, organ dysfunction parameters, and uteroplacental function indicators), making it impossible to retrospectively apply the revised diagnostic criteria. Consequently, due to these changes in diagnostic criteria and the lack of detailed clinical parameters in our dataset, the classification of preeclampsia would have been inconsistent both between institutions and across the study period.

Our study was further limited by the exclusion of stillbirths and miscarriages from the analysis. We recognize that these outcomes are crucial to understanding the full impact of different reproductive technologies. However, our dataset lacked important information on known influential factors, such as maternal thrombophilia, antiphospholipid antibodies, psychosocial factors, endometrial thickness at embryo transfer, and male infertility factors. Future studies should aim to include these data to provide a more comprehensive analysis of reproductive outcomes including stillbirths and miscarriages.

Our study investigated the safety profile of FET and FreshET in comparison with that of non‐ART treatments among infertile women. We found that the risk of neonatal anomalies was not significantly different between the non‐ART, FET, and FreshET groups after adjusting for confounding factors. However, FET was associated with a higher risk of obstetric complications. These findings suggest the need for careful management of FET cases and further research to improve treatment protocols and reduce risks. To ensure neonatal and maternal safety, ART and perinatal facilities must share information on the risks associated with different ART treatment protocols. Moreover, the continuous and comprehensive tracking of neonatal and maternal outcomes is essential. This study highlights the importance of establishing a seamless and continuous information sharing system between ART and perinatal facilities to optimize patient care and safety.

## CONFLICT OF INTEREST STATEMENT

Author R.S. was an employee of CureApp, Inc. during the study and reports grants from Osake‐no‐Kagaku Foundation. R.S. also received personal fees from Otsuka Pharmaceutical Co., Ltd., Nippon Shinyaku Co., Ltd., Takeda Pharmaceutical Co., Ltd., and Sumitomo Pharma Co., Ltd. outside the submitted work. These funding sources had no role in the design, practice or analysis of this study. Additionally, R.S. has patents JP2022049590A, US20220084673A1, JP2022178215A, and JP2022070086, and a pending patent application JP2023074128A. The other authors have no conflicts of interest.

## ETHICS STATEMENT

All procedures were performed in accordance with the ethical standards of the relevant committees on human experimentation (institutional and national) and the Helsinki Declaration of 1964 and its later amendments. This study was conducted in accordance with the principles of the Declaration of Helsinki and approved by the institutional review board of Okayama Couple's Clinic (approval no. 2022–12). During the informed consent process at each stage of fertility treatment, patients were informed that their anonymized treatment data would be used for research purposes, and written consent for such use was obtained. In addition to obtaining written consent, an opt‐out approach was employed to ensure transparency and provide patients with the opportunity to decline participation. Detailed information regarding the study was made publicly available on the clinic's website and through informational materials displayed within the clinic. Patients were given sufficient time to review the information and the option to opt‐out of having their data included in the study. The method of obtaining informed consent and the opt‐out approach for the use of patient data in retrospective studies were approved by the institutional review board. To protect patient privacy and confidentiality, all identifying information was removed before data analysis.

## References

[1] Pinborg A . Short‐ and long‐term outcomes in children born after assisted reproductive technology. BJOG. 2019;126(2):145–148. 30120870 10.1111/1471-0528.15437

[2] Warner L , Jamieson DJ , Barfield WD . CDC releases a National Public Health Action Plan for the detection, prevention, and Management of Infertility. J Women's Health. 2015;24(7):548–549. 10.1089/jwh.2015.5355 26133516

[3] Katagiri Y , Jwa SC , Kuwahara A , Iwasa T , On M , Kato K , et al. Assisted reproductive technology in Japan: a summary report for 2021 by the ethics Committee of the Japan Society of obstetrics and gynecology. Reprod. Med Biol. 2024;23:e12552. 10.1002/rmb2.12552 PMC10757097 38163009

[4] Pinborg A , Henningsen AA , Loft A , Malchau SS , Forman J , Andersen AN . Large baby syndrome in singletons born after frozen embryo transfer (FET): is it due to maternal factors or the cryotechnique? Hum Reprod. 2014;29(3):618–627. 24413766 10.1093/humrep/det440

[5] Litzky JF , Boulet SL , Esfandiari N , Zhang Y , Kissin DM , Theiler RN , et al. Effect of frozen/thawed embryo transfer on birthweight, macrosomia, and low birthweight rates in US singleton infants. Am J Obstet Gynecol. 2018;218(4):433. 10.1016/j.ajog.2017.12.223 PMC5878119 29291410

[6] Davies MJ , Moore VM , Willson KJ , Van Essen P , Priest K , Scott H , et al. Reproductive technologies and the risk of birth defects. N Engl J Med. 2012;366(19):1803–1813. 22559061 10.1056/NEJMoa1008095

[7] Hansen M , Kurinczuk JJ , Milne E , de Klerk N , Bower C . Assisted reproductive technology and birth defects: a systematic review and meta‐analysis. Hum Reprod Update. 2013;19(4):330–353. 23449641 10.1093/humupd/dmt006

[8] Maheshwari A , Pandey S , Amalraj Raja E , Shetty A , Hamilton M , Bhattacharya S . Is frozen embryo transfer better for mothers and babies? Can cumulative meta‐analysis provide a definitive answer? Hum Reprod Update. 2018;24(1):35–58. 29155965 10.1093/humupd/dmx031

[9] Ishihara O , Araki R , Kuwahara A , Itakura A , Saito H , Adamson GD . Impact of frozen‐thawed single‐blastocyst transfer on maternal and neonatal outcome: an analysis of 277,042 single‐embryo transfer cycles from 2008 to 2010 in Japan. Fertil Steril. 2014;101(1):128–133. 24268706 10.1016/j.fertnstert.2013.09.025

[10] Wooldridge JM . Inverse probability weighted estimation for general missing data problems. J Econ. 2007;141(2):1281–1301.

[11] Wooldridge JM . Econometric analysis of cross section and panel data. second ed. Cambridge (MA): MIT Press; 2010. p. 1096.

[12] Aldrich JH , Nelson FD , Adler ES . The Linear Probability Model. Linear probability, logit, and probit models newbury park. California: Sage; 1984. p. 9–29.

[13] Ali MS , Prieto‐Alhambra D , Lopes LC , Ramos D , Bispo N , Ichihara MY , et al. Propensity score methods in health technology assessment: principles, extended applications, and recent advances. Front Pharmacol. 2019;18(10):973. 10.3389/fphar.2019.00973 PMC6760465 31619986

[14] Rubin DB . Using propensity scores to help design observational studies: application to the tobacco litigation. Health Serv Outcome Res Methodol. 2001;2(3):169–188.

[15] Mishra PC , Baveja R . Congenital malformations in the newborn—a prospective study. Indian Pediatr. 1989;26(1):32–35. 2788132

[16] Mezawa H , Tomotaki A , Yamamoto‐Hanada K , Ishitsuka K , Ayabe T , Konishi M , et al. Prevalence of congenital anomalies in the Japan environment and Children's study. J Epidemiol. 2019;29(7):247–256. 30249945 10.2188/jea.JE20180014 PMC6556438

[17] Garne E , Hansen AV , Birkelund AS , Andersen AMN . Major congenital anomalies in a Danish region. Dan Med J. 2014;61(6):A4825. 24947618

[18] Chukwubuike KE , Ozor I , Enyi N . Prevalence and pattern of birth defects in the two tertiary hospitals in Enugu, south East Nigeria: a hospital‐based observational study. Afr J Paediatr Surg. 2020;17:85. 33342840 10.4103/ajps.AJPS_59_20 PMC8051625

[19] Yu HT , Yang Q , Sun XX , Chen GW , Qian NS , Cai RZ , et al. Association of birth defects with the mode of assisted reproductive technology in a Chinese data‐linkage cohort. Fertil Steril. 2018;109(5):849–856. 29778384 10.1016/j.fertnstert.2018.01.012

[20] Hansen M , Kurinczuk JJ , Bower C , Webb S . The risk of major birth defects after intracytoplasmic sperm injection and in vitro fertilization. N Engl J Med. 2002;346(10):725–730. 11882727 10.1056/NEJMoa010035

[21] Hoorsan H , Mirmiran P , Chaichian S , Moradi Y , Hoorsan R , Jesmi F . Congenital malformations in infants of mothers undergoing assisted reproductive technologies: a systematic review and meta‐analysis study. J Prev Med Public Health. 2017;50(6):347–360. 29207452 10.3961/jpmph.16.122 PMC5717326

[22] Pelkonen S , Hartikainen AL , Ritvanen A , Koivunen R , Martikainen H , Gissler M , et al. Major congenital anomalies in children born after frozen embryo transfer: a cohort study 1995‐2006. Hum Reprod. 2014;29(7):1552–1557. 24812318 10.1093/humrep/deu088

[23] Maheshwari A , Raja EA , Bhattacharya S . Obstetric and perinatal outcomes after either fresh or thawed frozen embryo transfer: an analysis of 112,432 singleton pregnancies recorded in the human fertilisation and embryology authority anonymized dataset. Fertil Steril. 2016;106(7):1703–1708. 27678031 10.1016/j.fertnstert.2016.08.047

[24] Pinborg A , Loft A , Aaris Henningsen AK , Rasmussen S , Andersen AN . Infant outcome of 957 singletons born after frozen embryo replacement: the Danish National Cohort Study 1995‐2006. Fertil Steril. 2010;94(4):1320–1327. 19647236 10.1016/j.fertnstert.2009.05.091

[25] Roque M , Valle M , Sampaio M , Geber S . Obstetric outcomes after fresh versus frozen‐thawed embryo transfers: a systematic review and meta‐analysis. JBRA Assist Reprod. 2018;22(3):253–260. 29782139 10.5935/1518-0557.20180049 PMC6106638

[26] Sha T , Yin X , Cheng W , Massey IY . Pregnancy‐related complications and perinatal outcomes resulting from transfer of cryopreserved versus fresh embryos in vitro fertilization: a meta‐analysis. Fertil Steril. 2018;109(2):330–342.e9. 29331236 10.1016/j.fertnstert.2017.10.019

[27] Roque M , Haahr T , Geber S , Esteves SC , Humaidan P . Fresh versus elective frozen embryo transfer in IVF/ICSI cycles: a systematic review and meta‐analysis of reproductive outcomes. Hum Reprod Update. 2019;25(1):2–14. 30388233 10.1093/humupd/dmy033

[28] Saito K , Kuwahara A , Ishikawa T , Morisaki N , Miyado M , Miyado K , et al. Endometrial preparation methods for frozen‐thawed embryo transfer are associated with altered risks of hypertensive disorders of pregnancy, placenta accreta, and gestational diabetes mellitus. Hum Reprod. 2019;34(8):1567–1575. 31299081 10.1093/humrep/dez079

[29] Busnelli A , Schirripa I , Fedele F , Bulfoni A , Levi‐Setti PE . Obstetric and perinatal outcomes following programmed compared to natural frozen‐thawed embryo transfer cycles: a systematic review and meta‐analysis. Hum Reprod. 2022;37(7):1619–1641. 35553678 10.1093/humrep/deac073

